# Nutritional Indices of the Cotton Bollworm, *Helicoverpa armigera*, on 13 Soybean Varieties

**DOI:** 10.1673/031.010.1411

**Published:** 2010-09-13

**Authors:** B. Naseri, Y. Fathipour, S. Moharramipour, V. Hosseininaveh

**Affiliations:** ^1^ Department of Entomology, Faculty of Agriculture, Tarbiat Modares University, P.O. Box 14115-336, Tehran, Iran.; ^2^ Department of Plant Protection, College of Agriculture, University of Tehran, Karaj, Iran

**Keywords:** antibiosis, food consumption, insect weight, Noctuidae, soybean resistance

## Abstract

The effects of 13 soybean varieties (356, M4, M7, M9, Clark, Sahar, JK, BP, Williams, L17, Zane, Gorgan3, and DPX) on nutritional indices of the cotton bollworm, *Helicoverpa armigera* (Hübner) (Lepidoptera: Noctuidae), were determined at 25 ± 1° C, 65 ± 5% RH and a photoperiod of 16:8 L:D. Fourth instar larvae reared on Zane showed the highest efficiency of conversion of digested food (ECD) and approximate digestibility (AD) values (0.299 and 0.867, respectively) compared with other varieties. The lowest value of ECD and food consumed (FC) was on 356 (0.133 and 53.82 mg, respectively). The highest and lowest efficiency of conversion of ingested food (ECI) of fifth instar larvae (0.235 and 0.156, respectively) were on Zane and M4, respectively. The ECI and ECD values of whole larval instars were the highest on M7 (0.524 and 0.820, respectively) and lowest on Sahar (0.279 and 0.353, respectively). However, the highest and lowest value of consumption index (CI) was on M7 (7.351) and BP (3.462). Among the different varieties of soybean, the highest AD value was on M9 (0.858), and the lowest was on Zane (0.597). The results indicated that M4, Sahar, and JK were partially resistant to *H. armigera*.

## Introduction

The cotton bollworm, *Helicoverpa armigera* (Hübner) (Lepidoptera: Noctuidae), is a highly destructive polyphagous pest causing severe loss to many economically important crops, including soybean, in Iran (Farid 1986) and elsewhere in the world (Reddy et al. 2004; Subramanian and Mohankumar 2006; Mironidis and Savopoulou-Soultani 2008). It is a major pest for 181 cultivated and uncultivated plant species, distributed in 45 families in India (Manjunath et al. 1989), and it creates serious problems in tomato (Moral Garcia 2006), leguminous (Singh and Mullick 1997), cotton (Kranthi et al. 2002), and pigeonpea (Kumari et al. 2006). Every year, the larvae of this species cause substantial economic losses to cotton, corn, tomato, legumes, and vegetable crops (Liu et al. 2004). The outbreak of this pest has been attributed to the development of insecticide resistance and the use of broad spectrum insecticides, which are known to have an detrimental effect on populations of its natural enemies and nutritional and bioclimatic factors in host plants (Fitt et al. 1995; Naseri et al. 2009). Therefore, the present research has increasingly been carried out to identify alternative measures to chemical control.

The chemical composition of host plants significantly affects survival, growth, and reproduction of phytophagous insects (Bernys and Chapman 1994). Food consumption and utilization link plant attributes with insect performance (Slansky 1990). For polyphagous insects, the availability of different host plants plays an important role in triggering population outbreaks (Singh and Parihar 1988). Growth, development, and reproduction of insects are strongly dependent on the quality and quantity of food consumed (Scriber and Slansky 1981).

Of the tools of pest management, host plant resistance is important in terms of being both economically and environmentally acceptable. Therefore, as a method of controlling pest insects, host plant resistance is not only favorable to the environment, but also reduces expenses for growers (Li et al. 2004). The factors determining nutrient availability for growth and maintenance over a given period of development are the amount and type of food consumed and the efficiency with which is utilized (Barton Browne and Raubenheimer 2003).

Previously Naseri et al. (2009) examined life history and fecundity of *H. armigera* on different varieties of soybean. The data obtained in that study allowed for an estimate of two of the major factors determining the susceptibility of soybean varieties, the developmental time and fecundity of *H. armigera*. In this research, this work was extended, and the effects of different soybean varieties on nutritional indices of *H. armigera* were elucidated as other factors determining the susceptibility of the examined varieties to this pest. By combining the data from the earlier study and the findings of the current research, a comprehensive scheme for an integrated pest management program for *H. armigera* on soybean could be designed.

In spite of the economic importance of *H. armigera*, no information exists on the nutritional indices of this pest on different soybean varieties, although some related studies have been conducted on the effects of host plants, apart from soybean varieties, on nutritional indices of *H. armigera* (Ashfaq et al. 2003) and on growth and food consumption of *Heliothis zea* (Farrar and Kennedy 1987). Therefore, the present study provides new information on the nutritional indices of *H. armigera* on different soybean varieties.

## Materials and Methods

### Plant sources

Seeds of the 13 soybean (*Glycine max* (L.) Merrill) varieties, including 356 (Delsoy4210), M4, M7, M9, Clark, Sahar, JK, BP, Williams, L17, Zane, Gorgan3, and DPX, were acquired from the Plant and Seed Modification Research Institute, Karaj, Iran. They were grown in the research field of Tarbiat Modares University in the suburbs of Tehran, Iran in May 2008. For this study, the leaves and pods of different soybean varieties were transferred to a growth chamber at 25 ± 1° C, 65 ± 5% RH, and a photoperiod of 16:8 L:D and used for feeding of first larval instars (leaves) and second to fifth larval instars (pods).

### Laboratory colony

Originally, *H. armigera* specimens were collected from cotton fields in the Moghan region located in northwest Iran in July 2007. Stock culture was initiated on an artificial diet (Twine 1971; Naseri et al. 2009) in a growth chamber at 25 ± 1°, 65 ± 5% RH, and a photoperiod of 16:8 L:D.

### Experiments

Newly hatched larvae were collected from the stock culture and divided into four replicates (10 larvae in each) and transferred into plastic containers (diameter 16.5 cm, depth 7.5 cm) with a hole covered by a fine mesh net for ventilation, containing the fresh leaves of each examined plant. The petioles of detached leaves were inserted in water-soaked cotton to maintain freshness. Nutritional indices were determined using second to fifth instars as they were more easily measurable than the first instar. A fine camel's hair brush was used to transfer the younger larvae. First instar larvae were reared in groups until the third instar, after which they were separated into individual plastic tubes (diameter 3 cm, depth 5 cm) to prevent cannibalism. Fifth instar larvae were kept in the above-described tubes for pre-pupation and pupation.

A gravimetric technique was used to determine weight gain, food consumption, and feces produced. Nutritional indices were measured on the dry weight basis. After measuring the weight of the second instar larvae, they were introduced on the pods of different soybean varieties, and the weights of the larvae were recorded daily before and after feeding until they finished feeding and reached the pre-pupal stage. The pre-pupa, pupa, and adults from the larvae reared on each variety were weighed as well. The initial fresh pods and the pods and feces remaining at the end of each experiment were weighed daily. The quantity of food ingested was determined by subtracting the diet remaining at the end of each experiment from the total weight of diet provided. The weight of feces produced by the larvae fed on each soybean variety was recorded daily. To find the dry weights of the pods, feces, and larval to adult stages, extra specimens (20 specimens for each) were weighed, oven-dried (48 hours at 60° C), and then re-weighed to establish a percentage of their dry weight. The forewing area of *H. armigera* adults reared on each soybean variety during its immature stages was also measured.

The following formulae were used according to Waldbauer (1968) to calculate CI (consumption index), AD (approximate digestibility), ECI (efficiency of conversion of ingested food) and ECD (efficiency of conversion of digested food):

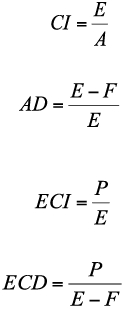

where, *A* = mean dry weight of insect over unit time, *E* = dry weight of food consumed, *F* = dry weight of feces produced, and *P* = insect dry weight gain.

### Data analysis

Nutritional indices of *H. armigera* reared on different soybean varieties were analyzed with one way ANOVA using the statistical software Minitab 14 to determine the similarities or significant differences. Statistical differences among the means were evaluated using the least significant differences (LSD) test at α = 0.05. Data were checked for normality prior to analysis.

**Table 1.**
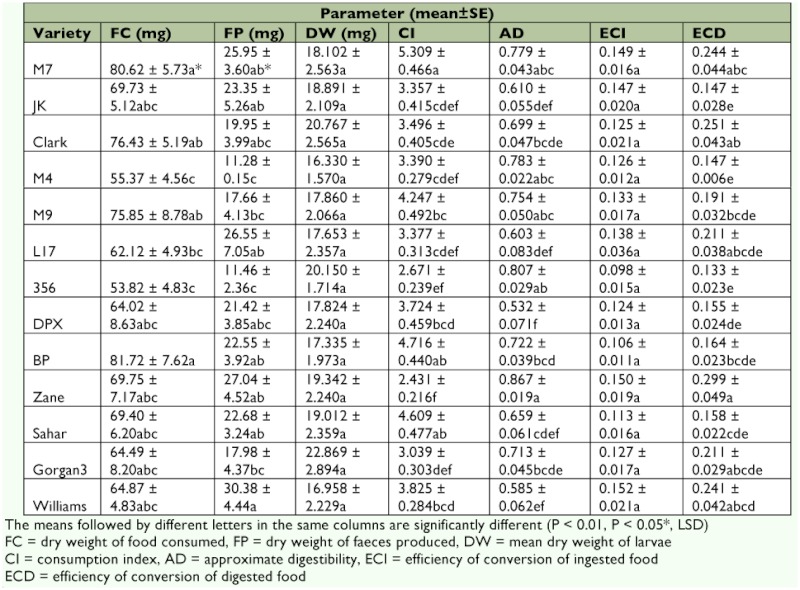
Nutritional indices of fourth instar larvae of *Helicoverpa armigero* on different soybean varieties

A dendrogram of soybean varieties based on nutritional indices of *H. armigera* overall 2^nd^ to 5^th^ instars (second instar + third instar + fourth instar + fifth instar larvae), herein whole larval instars, reared on different varieties of soybean was constructed after cluster analysis by Ward's method using SPSS 16.0 statistical software.

## Results

The results of the nutritional indices of fourth instar, fifth instar, and whole larval instars of *H. armigera* are provided in Tables 1, 2, and 3. Nutritional indices of fourth instar larvae of *H. armigera* were significantly different on soybean varieties (p < 0.05). The larvae reared on Zane showed the highest value of ECD (0.299 ± 0.049) (*F* = 2.42; df = 12, 150; p < 0.01) and AD (0.867 ± 0.019) (*F* = 4.06; df = 12,158; p < 0.01) compared with those reared on the other varieties. The lowest value of ECD and food consumed (*F* = 1.94; df = 12, 179; p < 0.05) was on 356 (0.133 ± 0.023 and 53.82 ± 4.83 mg, respectively). The larvae fed on DPX had the lowest AD value (0.532 ± 0.071). The CI of larvae reared on M7 showed the highest value (5.309 ± 0.466). However, the lowest value of this parameter (2.431 ± 0.216) was observed on variety Zane (*F* = 5.29; df = 12, 164; p < 0.01). Data in Table 1 indicates that there were no significant differences between larval weight (*F* = 0.58; df = 12, 152; p = 0.858) and ECI (*F* = 1.00; df = 12, 152; p = 0.448) of *H. armigera* on soybean varieties.

**Table 2.**
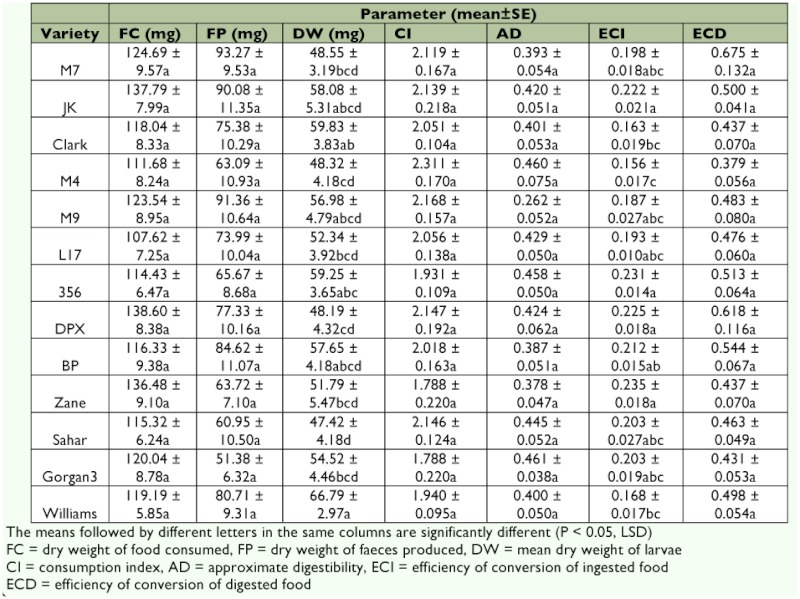
Nutritional indices of fifth instar larvae of *Helicoverpa armigera* on different soybean varieties

The larval weight (*F* = 2.16; df = 12, 365; p < 0.05) and ECI (*F* = 1.93; df = 12, 179; p < 0.05) of fifth instar *H. armigera* were found to be significantly different based on the soybean varieties on which individuals were reared. However, no significant difference was observed on the other estimated parameters of the pest on soybean varieties. The highest and lowest ECI values of *H. armigera* (0.235 ± 0.018 and 0.156 ± 0.017, respectively) were on Zane and M4, respectively. The larval weight of *H. armigera* showed significant difference, being heaviest on Williams (66.79 ± 2.97 mg) and lightest on Sahar (47.42 ± 4.18 mg).

The results presented in Table 3 for whole larval instars showed no significant difference for feces produced (*F* = 1.42; df = 12, 39; p = 0.198) and larval weight (*F* = 1.92; df = 12, 39; p = 0.063). The ECI (*F* = 3.46; df = 12, 39; p < 0.01) and ECD (*F* = 3.67; df = 12, 39; p < 0.01) values of the whole larval instars were the highest on M7 (0.524 ± 0.040 and 0.820 ± 0.046, respectively) and lowest on Sahar (0.279 ± 0.068 and 0.353 ± 0.119, respectively). However, the highest and lowest values of CI were on M7 (7.351 ± 0.958) and BP (3.462 ± 0.152), respectively (*F* = 2.52; df = 12, 39; p < 0.05). Among the different varieties of soybean, the highest value of AD was on M9 (0.858 ± 0.064), and the lowest was on Zane (0.597 ± 0.039) (*F* = 3.39, df = 39, p < 0.01).

Different soybean varieties showed no significant effect on the adults' weight and forewing area of *H. armigera*. However, the wet and dry weights of the pre-pupa (*F* = 2.82; df = 12, 184; p < 0.01) and pupa (*F* = 5.01; df = 12, 204; p < 0.01) were affected significantly by the variety of soybean (Table 4). Pre-pupa and pupa of larvae reared on Clark were heavier than those of larvae reared on other varieties tested.

**Table 3.**
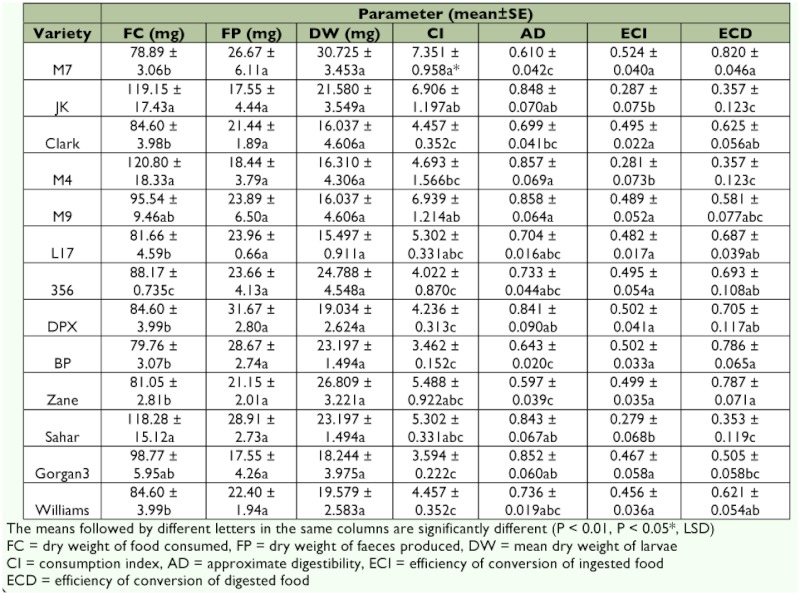
Nutritional indices of whole larval instars of *Heiicoverpa armigera* on different soybean varieties

### Cluster analysis

**Figure 1.**
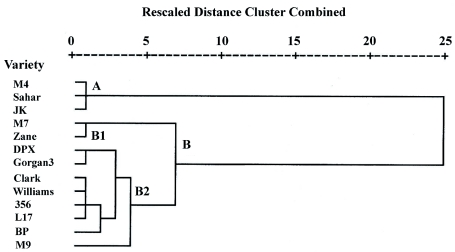
Dendrogram of different soybean varieties based on nutritional indices of *Helicoverpa armigera* reared on different soybean varieties. High quality figures are available online.

A dendrogram based on nutritional indices of *H. armigera* whole larval instars reared on different varieties of soybean is shown in Figure 1. The dendrogram shows two distinct clusters labelled A and B (including subclusters B1 and B2). Different varieties were grouped within each cluster based on the comparison of the nutritional indices of *H. armigera* reared on the varieties. Cluster A included M4, Sahar, and JK as a partially resistant group; cluster B consisted of subclusters B1 (M7 and Zane) as a susceptible group and B2 (DPX, Gorgan3, Clark, Williams, 356, L17, BP, and M9) as an intermediate group.

**Table 4.**
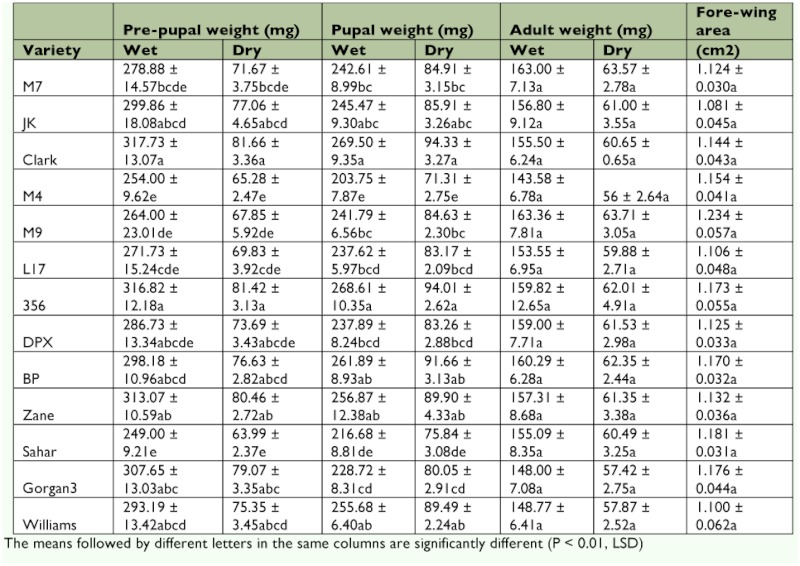
The mean (±SE) body weights of pre-pupa, pupa and adult stages and fore-wing area of *Helicoverpa armigera* on different soybean varieties

## Discussion

Using resistant varieties is one of the core strategies of an integrated pest management program, and secondary substances of plants or allelochemicals play a major role in plant resistance to pests (Wilson and Huffaker 1976). The use of soybean resistant to insects offers an important tool in integrated pest management (Endo et al. 2007). Differences in allelochemical concentrations between host plant varieties can affect an insect's performance as larva (Martin and Pulin 2004). The ability of an organism to convert nutrients, especially protein, will positively influence its growth and development (Sogbesan and Ugwumba 2008).

Significant differences were found within the nutritional indices, especially ECI and ECD values, of *H. armigera* reared on different soybean varieties, suggesting that the varieties have different nutritional value. Among nutritional indices, ECI may vary with the digestibility of food and the proportional amount of the digestible portion of food which is converted to body mass and metabolized for energy needed for vital activity (Abdel-Rahman and Al-Mozini 2007). ECI is an overall measure of an insect's ability to utilize the food ingested for growth and development, and ECD is a measure of the efficiency of conversion of digested food into growth (Nathan et al. 2005). Change in ECD also indicates the overall increase or decrease of the proportion of digested food metabolized for energy. Therefore, no change in ECI and ECD values indicate that ingested secondary biochemicals do not exhibit any chronic toxicity (Koul et al. 2004).

No significant difference was observed on the nutritional indices of the fifth instar except for the larval weight and ECI. However, the nutritional indices of the fourth instar larvae of *H. armigera* were significantly different depending on the type of soybean variety. Therefore, the data generated for the fourth and fifth instars are not consistent with each other. This is due to the fact that the nutritional requirements of an insect change during development, and such changes are typically reflected in changes of food consumption and feeding behavior (Barton Browne 1995). In larvae, the nutritional requirements over different developmental periods are positively correlated with growth over that period, since growth is directly based on nutrient input. It is likely due to the fact that nutritional requirements would be positively correlated with the mass of the insect (Schroeder 1981; Phillipson 1981). According to Barton Browne and Raubenheimer (2003), total consumption in the fifth instar of *H. armigera* reared on a navy bean-based diet was about 3.5 times greater than in the fourth instar, mainly due to the greater rate of ingestion. Furthermore, the results of life table studies of *H. armigera* on different host plants (Liu et al. 2004) showed that the fourth instar larvae reared on corn were the heaviest, while larvae reared on tomato and tobacco were the lightest. However, the last instar larvae fed on cotton were heavier than those reared on other host plants. Another possible reason for this variation could be due to the age of larva in a particular stadium at the time of weighing. For instance, the weights of either fourth or fifth stadia are expected to be lower when the larvae are near to entering the next stadium (where the larva stops feeding before entering the next stadium) or have recently entered the next stadium (where it looses some water and the exuviae) as compared to larvae growing in the mid-part of any stadia.

Additionally, differences in physiological changes during penultimate and ultimate instar larvae are probably partially responsible for the differences in data generated for these two larval instars on soybean varieties. Juvenile hormone (JH) is one of the major controlling hormones in development changes such as molting and metamorphosis. Juvenile hormone also determines whether major changes will occur in internal organs; usually little or no changes in internal morphology occur between larval molts, but major changes occur during transformation into pupa or adult (Nation 2000). Physiological changes in the nervous system of the fifth instar cause cessation of feeding, induces wandering behavior, and metabolic changes that occur in the fat body. Because of such physiological and behavioral changes, the feeding period of the larvae was shorter in fifth instar than the fourth instar, and subsequently nutritional responses of these two larval instars were different.

The highest ECI value of *H. armigera* was on varieties Zane and M7, indicating that they were more efficient at the conversion of ingested food to biomass. As can be seen in Table 3, the larvae fed on the Sahar variety had the lowest value of ECD, which suggests that these larvae were apparently not as efficient in turning digested food into biomass. It is well known that the degree of food utilization depends on the digestibility of food and the efficiency with which digested food is converted into biomass (Batista Pereira et al. 2002). The reduction in dietary utilization suggests that reduction in nutritional values may be resulted from both behavioral and physiological effects (Nathan et al. 2005). The mean ECD value from this study of whole larval instars reared on different soybean varieties was higher than that reported by Wang et al. (2006) on an artificial diet (0.412 ± 0.012).

Among different varieties of soybean, the highest CI value of *H. armigera* was on variety M7, indicating that the rate of intake relative to the mean larval weight during the feeding period was the highest on this variety. The results for the AD value of fourth instar larvae of *H. armigera* fed on Clark (0.699 ± 0.047) and Sahar (0.659 ± 0.061) were nearly similar to those reported by Ashfaq et al. (2003) on *Sorghum vulgaris* Pers. (0.697) and *Gossypium hirsutum* L. var. NIAB-98 (0.662). Wang et al. (2006) noted that AD value of *H. armigera* was 0.214 ± 0.013 on an artificial diet.

According to the results of the cluster analysis, grouping within each cluster might be due to a high level of physiological similarity of soybean varieties, whereas the separate clusters might present significant variability in physiological characteristics between clusters. The results of the comparison of nutritional indices of *H. armigera* on different soybean varieties revealed that cluster A varieties were the least suitable and that subcluster B1 varieties were the most suitable host plants for *H. armigera*, while the varieties in subcluster B2 had an intermediate status.

The body weight is an important fitness indicator of insect population dynamics (Liu et al. 2004). Pupal weight can be an indirect, but easily measured, indicator of lepidopteran fitness (Leuck and Perkins 1972). The pupae produced by larvae reared on Sahar and M4 were lighter than that of pupae produced by larvae reared on the other varieties. This reinforces the suggestion that Sahar and M4 are more unsuitable host plants for *H. armigera* larvae than the others. Liu et al. (2004) showed that the pupal weight of *H. armigera*, which ranged from 167.1 ± 3.9 mg on tomato to 285.2 ± 4.2 mg on corn, was affected by different host plants. The present findings on the pupal weight of *H. armigera* reared on variety Zane (256.87 ± 12.38 mg) were similar to those reported by Liu et al. (2004) on common bean (257.1 ± 5.1 mg). Furthermore, the heaviest pupal weight of *H. armigera* was on variety Clark. According to an earlier study (Naseri et al. 2009), the larval period of *H. armigera* was the shortest on variety Clark, and also, the cluster analysis of that study revealed that the variety Clark was grouped within a susceptible cluster. In spite of the fact that a significant difference was found between the pupal weights of *H. armigera* on 13 soybean varieties, no significant differences were observed for adult weights.

The quality of larval food may affect the pupal and adult phenotypic characteristics. Obvious effects of larval diets are pupal distortions and wing malformations in the imago (Rosenthal and Dahlman 1975). The fecundity (number of eggs laid per female), longevity, and forewing area of lepidopteran adults are the most commonly used parameters for determining the effect of larval diet on the adult stage. In this study, adult weight and forewing area of adults reared on different soybean varieties was examined. Because no significant effects were found for the larval host plants (soybean varieties) on the adult size (forewing area), these effects likely have disappeared in the weight of adults. However, previous research (Naseri et al. 2009) showed significant effects on fecundity of *H. armigera* fed on different soybean varieties. Additionally, the insect's ability to store energy (e.g., pupal weight and lipids and glycogen levels) varied depending on the larval host plants (Liu et al. 2007). However, the effects of host plants on pupal weight, adult weight, and larval growth are independent of each other (Hwang et al. 2008).

The results of the present study suggested that M7 and Zane were more nutritive, and M4, Sahar and JK were less nutritive for *H. armigera* larvae than the others. The results related to M7 and Sahar (as suitable and unsuitable host plants, respectively) are in agreement with previous findings (Naseri et al. 2009). The results of that study on the life history and fecundity of *H. armigera* reared on the 13 soybean varieties indicated that the shortest development time, the lowest percentage mortality of immature stages, highest daily fecundity (eggs per reproduction day), and the total fecundity (eggs during reproduction period) were on variety M7, which is consistent with the current research regarding ECI and ECD values of whole larval instars on this variety. Cluster analysis of the previous study and the present experiment strongly demonstrate the susceptibility of M7 to *H. armigera* compared with the other varieties. Additionally, the dendrogram of soybean varieties of that study showed that variety Sahar was partially resistant due to longer development time, higher mortality, and lower development index of the immature stages on this variety, which is consistent with the results of cluster analysis of the present research on nutritional indices of *H. armigera* on 13 soybean varieties.

Analysis of nutritional indices can lead to the understanding of the behavioral and physiological basis of an insect response to host plants (Lazarevic and Peric-Mataruga 2003). Variation in the nutritional indices of the pest on different soybean varieties could be due to the result of differences in plant quality, either reflected by a difference in nutrients required by the pest or differences in the level of secondary biochemicals. The least suitability of some varieties as a host plant of *H. armigera* may be due to the presence of some secondary phytochemicals in these varieties acting as antixenotic and/or antibiotic agents or absence of primary nutrients essential for growth and development of *H. armigera*.

There are many factors affecting host suitability including nutrient content and secondary substances of the host and the capability of digestion and assimilation by an insect. For a better understanding of the insect-plant interaction, basic biochemical studies for the extraction and identification of phytochemicals, which adversly influence the build up of *H. armigera* populations on soybean are required. Through this research, the population dynamics of the pest may be determined on different host varieties and the information could be used to manage the pest population to below the economic injury level. Meanwhile, these results provide data for establishing suitable conditions for rearing *H. armigera*. For instance, mass culture methods could be improved by selecting host plants for rapid development, maximum survival, or high fecundity in order to use these individuals for mass rearing of natural enemies.

## Abbreviations

AD: approximate digestibility;
Cl: consumption index;
ECD: efficiency of conversion of digested food;
ECI: efficiency of conversion of ingested food
